# Benefits of early MR-Imaging in patients with acute spontaneous intracerebral hemorrhage: a retrospective study

**DOI:** 10.1186/s12883-024-03992-7

**Published:** 2024-12-20

**Authors:** Benedikt Grund, Anne Ebert, Vesile Sandikci, Eva Neumaier-Probst, Angelika Alonso

**Affiliations:** 1 Department of Neurology, Mannheim Center for Translational Neuroscience, Medical Faculty Mannheim, University of Heidelberg, Theodor-Kutzer-Ufer 1-3, Mannheim, 68167 Germany; 2 Department of Neuroradiology, Medical Faculty Mannheim, University of Heidelberg, Mannheim, Germany

**Keywords:** Intracerebral hemorrhage, Hemorrhagic stroke, Early magnetic resonance imaging, Neuroimaging

## Abstract

**Background:**

Neuroimaging plays a vital role in the diagnosis of intracerebral hemorrhage (ICH) and in identifying the underlying etiology for appropriate therapeutic approach. This study aims to determine the significance and potential advantages of using early magnetic resonance imaging (MRI) as a diagnostic tool for ICH.

**Methods:**

This retrospective study included 359 patients with ICH treated at the Department of Neurology, Mannheim University Hospital between January 2017 and December 2021. Patient characteristics, stroke severity and imaging procedures were descriptively analyzed. Factors associated with the choice of imaging modalities were evaluated. The etiology of hemorrhage was retrospectively analyzed using the existing data. We recorded the reassignment of ICH etiology by comparing the assessment after first sole review of CT scan and then subsequent MRI review. The overall rate of reassignments and the reassignments per CT-based initial etiology were analyzed.

**Results:**

In the sample of 359 patients with ICH (mean age 73.1 years, 55.4% male), patients receiving an additional MRI were significantly younger (*p* < .001) and were less severely affected by stroke (median NIHSS score 5 vs. 15, *p* < .001). MRI was performed significantly less frequently in patients who died during hospitalization (11.7% vs. 63.9%, *p* < .001). MRI led to a reassignment of ICH etiology in 48.2% of cases (80/166), uncovering unknown underlying causes in 69% of cases (49/71). Reassignment occurred most frequently in patients with a CT-based diagnosis of hypertensive ICH (18/50). The most frequent reassigned etiologies after MR imaging were cerebral amyloid angiopathy (CAA; 36 patients) and secondary hemorrhage of an ischemic stroke (30 patients).

**Conclusions:**

Early MR imaging in patients with ICH improves the determination of underlying etiology and the conception of an appropriate treatment approach, potentially contributing to better patient outcomes.

## Introduction

Spontaneous non-traumatic intracerebral hemorrhage (ICH) accounts for approximately 10 to 15% of all strokes [1], being the second most common subtype with an overall incidence of 26.47 per 100,000 people per year [2]. In contrast to the decreasing incidence of ischemic stroke, the occurrence of ICH remained constant in the period from 1980 to 2008 [3]. This consistency may be caused by an aging population and the more widespread use of oral anticoagulants (OAC) leading to an increased incidence of ICH related to antithrombotic drugs (20.9% of all ICH) counterbalancing the decreased incidence of hypertensive ICH due to public health improvements in blood pressure control [4–6].

There is a variety of different etiologies with arterial hypertension, cerebral amyloid angiopathy (CAA) and use of OAC being the leading causes of ICH in adults [7]. Different underlying causes of ICH are linked to varying prognoses and outcomes. Exemplarily, ICH while receiving anticoagulant treatment has an increased risk of hematoma expansion and rapid deterioration resulting in poor outcome [6].

Appropriate treatment also depends on the underlying etiology of ICH. Major elements to therapeutical management are blood pressure control (110-140mmHg as systolic target) and antagonization of OAC [8, 9]. Furthermore, neurosurgical interventions such as decompressive craniectomy, minimally invasive surgical hematoma evacuation and external ventricular drainage are used when indicated e.g. in rapidly deteriorating patients [10]. Etiology-specific treatment strategies in the acute phase include anticoagulation in parenchymal bleeding caused by cerebral venous thrombosis or surgical treatment of cavernomas or arterial vascular malformations [11, 12].

The first diagnosis of an ICH is usually made by performing a non-contrast cranial computed tomography (cCT) in patients with focal neurological deficits followed by a CT-angiography (CTA) to rule out arterial vascular malformations [13].

Because of its broad availability, rapidness, high diagnostic precision, and relatively low costs cCT is the imaging modality of choice to diagnose an ICH, even though magnetic resonance imaging (MRI) can as accurately detect an acute brain hemorrhage without radiation exposure [6, 14]. Instead, MRI is typically used in the subacute phase to identify the ICH etiology and to stratify the risk of recurrence, as it provides detailed information about anatomy, characteristics of the hemorrhage and vascular pathologies [10].

Hence, our study aims to retrospectively investigate the role of early MR-imaging in diagnosing and assigning an etiology to ICH in a patient sample observed from January 2017 to December 2021. By accurately identifying the underlying mechanism of hemorrhage through the incorporation of an additional MRI scan, we anticipate an improved attribution of etiology and thereby an optimized therapeutic approach.

## Methods

This retrospective study was approved by the Ethics Committee II of the University of Heidelberg - Medical Faculty Mannheim (reference number 2021 − 887). We screened all adult patients who were treated at the Department of Neurology, Mannheim University Hospital between January 2017 and December 2021 with an admission diagnosis of spontaneous nontraumatic ICH by ICD-10 codes (I61ff). Patients with underlying vascular malformation (arterio-venous malformation, angioma, ruptured aneurysm) were excluded if detected in the initial imaging. Further, patients who developed an ICH after treatment of an acute ischemic stroke (intravenous thrombolysis or thrombectomy) as well as patients who underwent immediate rescue operations were also excluded. The diagnosis of ICH was made using cranial computed tomography (cCT; Somatom Volume Zoom; Siemens Healthineers AG, Erlangen, Germany) and cranial magnetic resonance imaging (cMRI) as diagnostic tools. The choice of the imaging modalities was left to the attending physician’s discretion. Early MRI was defined as an imaging procedure within the index hospitalization, urgent MRI was defined as imaging procedure within 24 h of hospitalization. MRI was performed on a 1.5 T scanner (Magnetom Sonata or Avanto; Siemens Healthineers AG, Erlangen, Germany) or a 3 T scanner (Magnetom Trio; Siemens Healthineers AG, Erlangen, Germany) using a standardized protocol including transverse, coronal and sagittal localizing sequences followed by transverse oblique contiguous images with a slice thickness of 5 mm aligned with the inferior borders of the corpus callosum (applied on sequences 2–6), T1-weighted images, T2-weighted images, diffusion-weighted images (DWI), FLAIR images, T2*-weighted images; a 3D time-of-flight MRA. The Stroke Unit database and the electronic paper records of Mannheim University Hospital were used for the retrospective data collection. Besides basic demographic information such as age, sex, length of stay we recorded pre-existing conditions like previous strokes or ICH, anticoagulation, the premorbid modified Rankin Scale (mRS) and risk factors such as arterial hypertension, diabetes mellitus and atrial fibrillation. To measure the severity of strokes, the National Institute of Health Stroke Scale (NIHSS) and the Glasgow Coma Scale (GCS) score upon admission were assessed. We recorded number and modality of imaging analyses per patient. ICH etiology was assessed by two independent neurologically trained raters (BG and AA) and a senior neuroradiologist rater (ENP) considering personal review of images and the synopsis of the neuroradiological report, information of medication and patient history. Cases with discrepancies were re-reviewed by both raters and discussed until a consensus was reached. Hypertensive ICH was diagnosed if the ICH was located in the anterior basal ganglia, thalamus, brainstem or cerebellum, together with a history of arterial hypertension. Possible or probable CAA was diagnosed according to the Boston criteria 2.0 [15]. For CT-based diagnosis of CAA, the simplified Edinburgh criteria for lobar intracerebral hemorrhage associated with cerebral amyloid angiopathy were additionally applied [16]. Hemorrhagic transformation of an ischemic stroke was identified if MRI revealed additional DWI lesions, either remote or adjacent to the ICH, and/or if CTA or MRA detected an intracranial vessel occlusion within the vascular territory of the ICH. The diagnosis of an underlying cavernoma was based on the presence of the characteristic popcorn-sign on MRI.

**Fig. 1:**
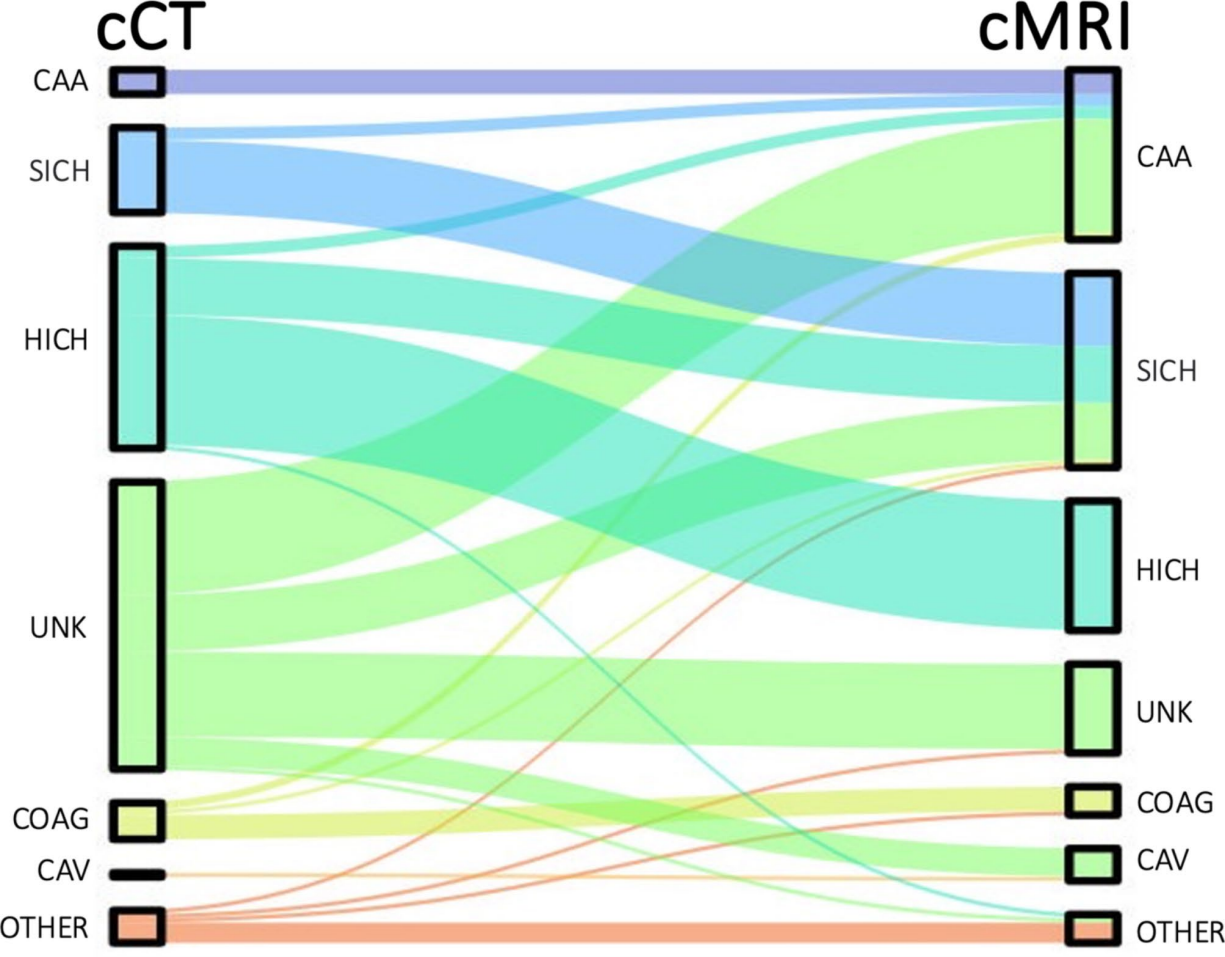
Sankey Plots visualizing the change of attributed etiology after performing an additional MRI scan. The graph was created using Microsoft PowerPoint^®^ 2022. CAA: Cerebral Amyloid Angiopathy, SICH: Secondary Hemorrhagic Transformation in Ischemic Stroke, HICH: Hypertensive ICH, UNK: Unknown, COAG: Coagulopathy, Cav: Cavernoma, Other: includes Neoplasm, Arteriovenous Malformation, Aneurysm, Vasculitis, Trauma, Sinus Vein Thrombosis, and others

For patients with both CT and MRI scans available, the most probable ICH etiology was first defined based on the CT scan, raters were blinded to the MRI results. As a second step, the most probable ICH etiology was determined based on the MRI scan. The transition in etiological diagnosis was recorded and graphically depicted as a Sankey plot (Fig. 1).

### Statistical analysis

IBM SPSS^®^ Statistics 27.0 was applied for statistical data analysis. For categorial and nominal variables like gender, risk factors, and pre-existing conditions, absolute and relative frequencies were recorded. Continuous metric variables such as age and length of stay were analyzed by calculating mean and standard deviation whereas the NIHSS and GCS scores upon admission were reported with median and interquartile range (IQR). Group comparisons for categorical variables were calculated using the chi-square test and for metric variables using the t-test for independent samples, while the Mann-Whitney U test was employed for independent group comparisons on ordinal or continuous variables. A p-value below 0.05 was considered significant.

## Results

In this retrospective study, 359 patients were included. The mean age was 73.1 ± 13.1 years. One hundred and ninety-nine patients (55.4%) were male. The length of stay averaged 13.7 ± 10 days. Arterial hypertension was the most frequent vascular risk factor (314 patients, 87.5%). In 141/359 patients (43.7%), a hypertensive crisis, defined as a blood pressure ≥ 180/120 mmHg, was recorded upon admission. Two hundred and ninety patients (80.8%) showed no relevant functional deficit prior to the ICH with a premorbid mRS from 0 to 2. Most patients (331/359, 92.2%) were admitted to stroke unit whereas initial admission to intensive care unit (ICU) occurred in only 6.1% (22/359).

327 patients (91.1%) received at least one CT scan, 198 (55.2%) one MRI scan and 166 (46.2%) both. Patients received a median of 2 CT scans (IQR 1–3) and 1 MRI scan (IQR 0–1). MRI was performed at a median of 1 day (IQR 1–4) after admission. One-hundred patients (50.5% of patients undergoing MR imaging) received an urgent MRI, 185 patients(93.4%) were examined within the first 7 days.

A previous ischemic stroke was documented in 54 patients (15%) and a previous ICH in 38 patients (10.6%). Sixty patients (16.7%) died during their stay.

A detailed summary of baseline characteristics is given in Table 1.

**Table 1:** Basic Demographic Information, Patient Characteristics and Clinical Details (n: 359)

Age, mean (SD), years	73.1 (13.1)
**Length of stay**,** mean (SD)**,** days**	13.7 (10.0)
**Sex**	
Female, n (%)	160 (44.6)
Male, n (%)	199 (55.4)
**Premorbid modified Rankin Scale**	
0–2, n (%)	290 (80.8)
3–5, n (%)	69 (19.2)
**Pre-existing condition**	
Arterial hypertension, n (%)	314 (87.5)
Diabetes mellitus, n (%)	86 (24.0)
Atrial fibrillation, n (%)	99 (27.6)
Previous ICH, n (%)	38 (10.6)
Previous stroke, n (%)	54 (15.0)
In-hospital death, n (%)	60 (16.7)
NIHSS upon admission, median (IQR)	8 (13)
GCS upon admission, median (IQR)	14 (3)
Anticoagulation at onset, n (%)	75 (20.9)
Reversal of anticoagulation, n (%)	49 (13.6)
Platelet inhibitors at onset, n (%)	113 (31.5)
Hypertensive crisis ≥ 180/120mmHg upon admission, n (%)	141 (43.7)
Surgical treatment, n (%)	31 (8.6)
DSA, n (%)	32 (8.9)
Mechanical ventilation (any timepoint), n (%)	29 (8.1)
Palliative treatment goal, n (%)	71 (19.8)
**ICH location**	
Supratentorial, n (%)	329 (91.6)
Infratentorial, n (%)	26 (7.2)
Both supra- and infratentorial, n (%)	4 (1.1)
Intraventricular, n (%)	107 (28.7)
**Admission**	
Stroke Unit	331 (92.2)
ICU	22 (6.1)
Regular ward/ death in emergency department	6 (1.7)
**Imaging**	
cCT, n (%)	327 (91.1)
cMRI, n (%)	198 (55.2)
cCT + cMRI, n (%)	166 (46.2)
cCT only, n (%)	161 (44.8)
cMRI only, n (%)	32 (8.9)
Number of cCT per patient, mean (SD)	2.3 (1.6)
Number of cMRI per patient, mean (SD)	0.64 (0.65)
Time to first MRI scan, median (IQR), days	1 (3)

ICH, intracerebral hemorrhage; NIHSS, National Institutes of Health Stroke Scale; GCS, Glasgow Coma Scale; DSA, digital subtraction angiography; ICU, intensive care unit; cCT, cranial computed tomography (consisting of native CT and CT-angiography); cMRI, cranial magnetic resonance imaging

Patients who received an MRI scan were significantly younger (70.7 ± 13.6 years) than those who only received a CT scan (76.0 ± 11.8 years, *p* < .001). There was also a significant difference in the severity of stroke. Patients with MRI had a median NIHSS score of 5 (IQR 2–10,75) and GCS score of 15 (IQR 14–15) upon admission while patients with CT only were more severely affected with a median NIHSS score of 15 (IQR 8–20; *p* < .001) and GCS score of 13 (IQR 9–15; *p* < .001). In line with these findings, MRI was performed significantly less frequently in patients who died during hospitalization (7/60, 11.7% vs. 191/299, 63.9%, *p* < .001). Antithrombotic treatment at onset and medical history were also associated with the choice of imaging modalities: Patients with oral anticoagulation at symptom onset were less likely to be examined with MRI (25/75, 33.3% vs. 173/284, 60.9%, *p* < .001). This was particularly the case for patients receiving reversal treatment for oral anticoagulation (12/49, 24.5% vs. 186/310, 60%, *p* < .001). Patients with hypertensive crisis at admission tended to be more frequently examined by CT only (72/141, 51.1% vs. 76/182, 41.8%, *p* = .06). Admission to stroke unit was associated with a significantly higher proportion of patients receiving an MRI (189/331, 57.1% vs. 9/28, 32.1%, *p* = .016), whereas patients with need of mechanical ventilation during the hospital course, as well as patients undergoing surgical ICH treatment, were less likely to be examined with MRI (6/29, 20.7% vs. 190/326, 58.3%, *p* < .001 and 11/31, 35.5% vs. 187/328, 57.0%, *p* = .024, respectively). Surgery was performed at a median of < 24 h (IQR < 24 h - <48 h). Of 22 patients receiving surgical therapy within 24 h of admission, only 4 patients were examined by MRI the same day whereas 4 of 7 patients with surgery > 48 h after admission had MR imaging. Lobar vs. subcortical location of ICH was not associated with the choice of imaging modality, while patients with infratentorial ICH tended to receive an MRI more frequently in comparison to supratentorial bleeding location (22/30, 73.3% vs. 176/329, 53.5%, *p* = .054). We observed no delays in treatment or adverse events in patients undergoing MR imaging.

In the subgroup of 166 patients who received both a CT and MRI scan, 80/166 patients (48.2%) had a reassignment of the attributed etiology after the additional information given by MRI. Figure 1 illustrates the shift in attributed etiologies.

The most prominent finding was the reduction of the proportion of patients with unknown ICH etiology from 42.8% (71/166) to 13.3% (22/166) by additional information through MR imaging. In these cases, the most frequent etiologies detected by MRI were cerebral amyloid angiopathy (CAA, *n* = 28), secondary hemorrhage of an ischemic stroke (*n* = 14) and cavernoma (*n* = 7). Moreover, 18 of 50 patients with CT-based diagnosis of a hypertensive ICH were assigned to another etiology after MR imaging, with CAA (*n* = 3) and secondary hemorrhage of an ischemic stroke (*n* = 14) being the most frequent corrected etiologies. In most patients with CT-based diagnosis of a secondary hemorrhage of an ischemic stroke the diagnosis was confirmed by MRI (*n* = 18). Still, in 3/21 (14.3%) of these patients, MRI revealed an underlying CAA. In contrast, the diagnosis of CAA based on a synopsis of CT (possible CAA according to Boston criteria 2.0 in 6/6; simplified Edinburgh high risk category in 3/6, medium risk category in 2/6, low risk category in 1/6) and medical history was unchanged by additional information through MRI in all cases (*n* = 6). Rare ICH etiologies detected by MRI were Wernicke encephalopathy (*n* = 1) and hyperperfusion syndrome after carotid thrombendarterectomy (*n* = 1).

## Discussion

MRI is not commonly used as a primary survey for ICH due to cost, availability, and the potential for increased risk to the patient from prolonged imaging time. However, MRI can be useful in understanding the underlying etiology of ICH and in determining the risk of recurrence by providing detailed information about anatomy and characteristics of the hemorrhage, which can inform secondary prevention strategies [10, 13]. In our study this usefulness is reflected in a significant change of 48.2% in the etiologies of ICH after performing an additional MRI. This change exceeds the diagnostic yield observed in other studies assessing the utility of early MRI in diagnosing ICH. Exemplarily, the diagnostic category or confidence changed in 15/67 patients (22%), in 27/70 patients (39%), and in 17/47 patients (36%) in the cited retrospective studies [14, 17, 18]. This is further highlighted by a recent meta-analysis evaluating the detection rate of secondary lesions on brain MRI among 1147 patients with spontaneous ICH, which reported a pooled detection rate of only 11% [19]. This meta-analysis also includes a related retrospective cohort study by the same authors, revealing a detection rate of secondary lesions as low as 5.2%. However, their definition of secondary lesions was limited to brain tumors, secondary hemorrhage of an ischemic stroke, cerebral venous sinus thrombosis, and underlying vascular malformations like cavernomas. They did not account for cerebral microbleeds or markers of small vessel disease, which are critical for diagnosing CAA [19]. This limitation likely contributed to the lower diagnostic yield of MRI reported in their study and meta-analysis.

Another notable strength of our study is its reliance on a large sample with a high proportion of patients with MRI of more than 50% and therefore larger subgroups of etiologies, while still allowing a comparison with the patient subsample only receiving a CT. In a retrospective study analyzing the utility of early MRI in ICH patients, Chalouhi and colleagues report MRI prior to discharge as standard in all patients with CT-detected ICH which differs from the protocols of many institutions where the decision of obtaining an additional MRI scan is made on a case-by-case basis as it is reflected in our study [20]. Unfortunately, imaging analysis was confined to detection of structural lesions and vessel malformations, but did not include hypertensive microhemorrhages, amyloid deposits or restricted diffusion around the hemorrhage, as it focused on neurosurgical relevance in the acute setting. However, the latter are key findings in MRI for superior identification of ICH etiology compared to CT, since they indicate an underlying CAA or secondary hemorrhage of an ischemic stroke [13]. This is reflected in our study, in which 36 patients had a change in their diagnosis to CAA and 30 patients to secondary hemorrhage of an ischemic stroke after receiving an MRI scan, making them the most frequently changed to etiologies together with cavernoma. These findings are in line with observations by Wijman et al., where the highest yield of MRI was in patients with ICH related to CAA and hemorrhagic transformation of an ischemic stroke, and by Dystewski et al. with ICH caused by CAA and cavernous angioma being the most frequent found etiologies by MRI [14, 17].

Because of its high accuracy in identifying cerebral microbleeds, cortical superficial siderosis, convexal subarachnoid hemorrhage, and white matter lesions, MRI is the pivotal method for in-vivo diagnosis of CAA within the Boston criteria (v2.0) framework [15]. A CT-based implementation of the Boston criteria lacks specificity as the transition from possible to probable CAA requires MR-based features as mentioned above. In contrast, the simplified Edinburgh CT criteria for lobar intracerebral hemorrhage associated with cerebral amyloid angiopathy showed a high specificity of 87.1% at a low sensitivity of 58.5% for the high risk category, but low specificity (47.4%) at high sensitivity (80.9%) for the low risk category [21]. This dilemma precludes the use of CT as reliable tool for the diagnosis of CAA.

Emergency management of spontaneous ICH is largely independent of the etiology and is based on blood pressure and coagulation management to prevent hematoma enlargement, complemented by surgical intervention in selected cases according to current guidelines [6]. However, a change in etiology can greatly influence therapy decisions in the subacute setting and in the long term: Patients with CAA are at a higher annual risk (7.4%) of recurrent ICH than patients with non-CAA-related ICH (1.1%), thus affecting the patient’s prognosis [22]. Furthermore, the safety of (re-)starting anticoagulation in patients with hemorrhage due to CAA is uncertain as antithrombotic strategies increase the relative risk of ICH, potentially outweighing the reduced risk of thromboembolism [7, 23]. In addition, there are multiple studies suggesting an increased risk of thrombolysis-related ICH in patients with CAA [24].

While the benefit of identifying CAA patients is obvious, also patients with deep ICH gain from MR imaging in the acute phase: 14 of 50 (28%) patients with CT-based diagnosis of hypertensive ICH were found to have secondary hemorrhage of an ischemic stroke. To identify patients with secondary hemorrhage of an ischemic stroke, an early MRI with its diffusion-weighted imaging is considered the key method for reliably detecting an acute ischemia. However, in a subacute setting the visibility of DWI lesions diminishes as ADC pseudonormalization occurs after 7 to 15 days, making it crucial to promptly perform an MRI scan [25]. As therapeutic consequence of the diagnosis, a secondary prophylaxis involving a combination of ASA and statins should be implemented, while conducting a comprehensive investigation of the underlying cause of ischemia [26].

Cavernous malformations, otherwise challenging to characterize on CT and usually not visible on DSA, have a characteristic presentation on MRI known as the mulberry or popcorn sign: a hypointense ring caused by hemosiderin deposits around a heterogeneous core in T2-weighted gradient-echo sequences [13, 27]. For cavernomas that have become symptomatic due to bleeding, surgical excision should be considered depending on the accessibility and risk of rebleeding [28].

Upon examination of the patient population with spontaneous ICH that received less MRI scans, it was observed that they were older and more severely affected (potentially being a confounder), highlighting the need to consider the trade-off between risk, cost, and benefits when deciding which patients should receive additional MRI scans, especially considering the patient’s prognosis. Based on the findings of our study, it may be beneficial for all patients to undergo routine MRI scans. Considering that in our cohort patients examined exclusively by CT were older on average and the incidence of CAA increases with age, several diagnoses of CAA might have been missed, with consequences for antithrombotic treatment decisions. Furthermore, it is not advisable to rely on a hypertensive crisis upon admission to decide on the need for an additional MRI scan, as our analysis indicates that more than one third of putative hypertensive intracerebral hemorrhages had a shift in their etiology. Also, oral anticoagulation at symptom onset should not be the sole factor for the choice of imaging modality, as it is crucial to confirm or rule out CAA for further treatment.

We have to acknowledge that certain constellations might call for CT as the fastest imaging modality to allow for timely intervention in life-threatening situations. In addition, patients with life-threatening ICH might require constant monitoring and might be too unstable for MR imaging. These constellations are reflected in our finding that the vast majority of patients undergoing surgery did so within less than 24 h of admission, and only a small minority of these was examined with MRI. In addition, the costs of MR imaging exceed the costs of CT imaging. However, data on cost-effectiveness with consideration of optimized patient management due to MR imaging information are lacking. Moreover, MR imaging is not available across the board, and availability of MRI is still no precondition for certified stroke units according to the certification criteria of the German Stroke Society. With the advancement of MRI technology in terms of faster scan times, higher precision, and wider availability, MRI may become a more viable option for ICH diagnosis, adding to its already existing strengths of providing detailed information about the characteristics of the hemorrhage and the underlying cause.

### Limitations

Retrospective studies rely on the quality of existing records. Hence, inadequate documentation can cause errors or bias which should be considered when interpreting the results of this study. Although the ICH etiology was attributed by three independent raters using all the available patient information, a gold standard based on postmortem brain pathology would have provided a more valid assessment of etiology. Another source of potential bias is the choice of the imaging modalities, which was left at the physician’s discretion. The decision for or against MRI can further be dependent on the availability of imaging modality, time constraints, presence of metallic implants and cost-benefit. This bias in selection of imaging modality might translate into a bias in detected etiologies. Furthermore, our study is limited by the exclusion of patients with underlying vascular malformations as their condition was diagnosed at initial CTA and immediately addressed by surgical treatment. Similarly, patients who underwent immediate rescue operations were also excluded. In these cases, a tissue sample was obtained during surgery making MR-imaging for determining the etiology of minor importance.

## Conclusion

In summary, the findings of this study indicate that early MR imaging may provide additional clinical benefits for patients with spontaneous ICH as it accurately identifies the underlying cause and thereby determines the optimal therapeutic approach, potentially resulting in improved patient outcomes. However, as there is a lack of prospective studies and randomized controlled trials regarding the utility of early MRI in patients with spontaneous ICH, further investigation is necessary.

## Data Availability

The datasets generated and analyzed during the current study are not publicly available due to privacy and confidentiality concerns, as they contain sensitive patient information but are available from the corresponding author on reasonable request.

## Abbreviations

ASA: acetylsalicylic acid
CAA: cerebral amyloid angiopathy
cCT: cranial computed tomography
CTA: CT-angiography
DSA: digital subtraction angiography
DWI: diffusion-weighted imaging
GCS: Glasgow Coma Scale
ICH: spontaneous non-traumatic intracerebral hemorrhage
ICU: intensive care unit
IQR: interquartile range
MRA: magnetic resonance angiography
MRI: magnetic resonance imaging
mRS: modified Rankin Scale
NIHSS: National Institute of Health Stroke Scale
OAC: oral anticoagulants
